# Accounting for preferential sampling in species distribution models

**DOI:** 10.1002/ece3.4789

**Published:** 2018-12-26

**Authors:** Maria Grazia Pennino, Iosu Paradinas, Janine B. Illian, Facundo Muñoz, José María Bellido, Antonio López‐Quílez, David Conesa

**Affiliations:** ^1^ Instituto Español de Oceanografía Centro Oceanográfico de Vigo Vigo Spain; ^2^ Departament ďEstadística i Investigació Operativa Universitat de València Valencia Spain; ^3^ Ipar Perspective Asociación Sopela Spain; ^4^ School of Mathematics and Statistics Centre for Research into Ecological and Environmental Modelling (CREEM) University of St Andrews St Andrews UK; ^5^ Instituto Español de Oceanografía Centro Oceanográfico de Murcia Murcia Spain

**Keywords:** Bayesian modelling, integrated nested Laplace approximation, point processes, species distribution models, stochastic partial differential equation

## Abstract

Species distribution models (SDMs) are now being widely used in ecology for management and conservation purposes across terrestrial, freshwater, and marine realms. The increasing interest in SDMs has drawn the attention of ecologists to spatial models and, in particular, to geostatistical models, which are used to associate observations of species occurrence or abundance with environmental covariates in a finite number of locations in order to predict where (and how much of) a species is likely to be present in unsampled locations. Standard geostatistical methodology assumes that the choice of sampling locations is independent of the values of the variable of interest. However, in natural environments, due to practical limitations related to time and financial constraints, this theoretical assumption is often violated. In fact, data commonly derive from opportunistic sampling (e.g., whale or bird watching), in which observers tend to look for a specific species in areas where they expect to find it. These are examples of what is referred to as *preferential sampling*, which can lead to biased predictions of the distribution of the species. The aim of this study is to discuss a SDM that addresses this problem and that it is more computationally efficient than existing MCMC methods. From a statistical point of view, we interpret the data as a marked point pattern, where the sampling locations form a point pattern and the measurements taken in those locations (i.e., species abundance or occurrence) are the associated marks. Inference and prediction of species distribution is performed using a Bayesian approach, and integrated nested Laplace approximation (INLA) methodology and software are used for model fitting to minimize the computational burden. We show that abundance is highly overestimated at low abundance locations when preferential sampling effects not accounted for, in both a simulated example and a practical application using fishery data. This highlights that ecologists should be aware of the potential bias resulting from preferential sampling and account for it in a model when a survey is based on non‐randomized and/or non‐systematic sampling.

## 1 INTRODUCTION

An increasing interest in Species distribution models (SDMs) for management and conservation purposes has drawn the attention of ecologists to spatial models (Dormann et al., 2007). SDMs are relevant in theoretical and practical contexts, where there is an interest in, for example, assessing the relationship between species and their environment, identifying and managing protected areas and predicting a species’ response to ecological changes (Latimer, Wu, Gelfand, & Silander, 2006). In all these contexts, the main issue is to link information on the abundance, presence/absence, or presence only of a species to environmental variables to predict where (and how much of) a species is likely to be present in unsampled locations elsewhere in space.

In studies of species distribution, collecting data on the species of interest is not a trivial problem ([Kery et al., 2010). With the exception of a few studies (Thogmartin, Knutson, & Sauer, 2006), SDMs frequently rely on opportunistic data collection due to the high cost and time consuming nature of sampling data in the field, especially on a large spatial scale (Kery et al., 2010). Indeed, it is often infeasible to collect data based on a well‐designed, randomized, and/or systematic sampling scheme to estimate the distribution of a specific species over the entire area of interest (Brotons, Herrando, & Pla, 2007). Hence, various types of opportunistic sampling schemes are commonly used. As an example, studies on sea mammals commonly resort to the affordable practice of sampling from recreational boats (so‐called platforms of opportunity), whose bearings are neither random nor systematic (Rodríguez, Brotons, Bustamante, & Seoane, 2007). Similarly, bird data are often derived from online databases such as *eBird*, which make available locations of birds sighted by bird‐watchers, who tend to visit habitats suitable for interesting species (https://ebird.org/). Also, in the context of fishery ecology, fishery‐dependent survey data are often derived from commercial fleets tend to be readily available for analysis. However, the fishing boats naturally tend to fish in locations where they expect a high concentration of their target species (Vasconcellos & Cochrane, 2005).

All these types of opportunistically collected data tend to suffer from a specific complication: The sampling scheme that determines sampling locations is not random, and hence not independent of the response variable of interest, for example, species abundance (Conn, Thorson, & Johnson, 2017; Diggle, Menezes, & Su, 2010). However, SDMs typically assume, if only implicitly, that sampling locations are not informative and that they have been chosen independently of what values are expected to be observed in a specific location. This assumption is typically violated for opportunistic data resulting in preferentially sampled data, collected in locations that were deliberately chosen in areas where the abundance of the species of interest is thought to be particularly high or low. This violation leads to biased estimates and predictions (Diggle et al., 2010).

Consequently, biased estimation and predictions of species distribution lead to badly informed decision making and to inefficient or in appropriate management of natural resources (Conn et al., 2017; Diggle et al., 2010; Dinsdale & Salibian‐Barrera, 2018). This paper seeks to address preferential sampling in the context of fisheries ecology, where this issue is particularly relevant since the identification and management of sensitive habitats (e.g., through marine protected areas, nurseries, high‐discard locations) is a common conservation tool used to sustain the long‐term viability of species populations.

Diggle et al. (2010) suggest a modeling approach that accounts for preferential sampling using likelihood‐based inference with Monte Carlo methods. However, the resulting approach can be computationally intensive, as the authors recognize in their reply to the issues raised on the discussion of their paper, which implies that it is quite difficult to use in practical situations, especially when the objective of the analysis is to predict into in extended areas. This is of significant concern as prediction is often the main objective of SDMs and preferential sampling issues are by their very definition a practical problem that needs to be addressed in a form that makes them accessible to users.

As Rue, Martino, Mondal, and Chopin (2010) indicate in the discussion on Diggle et al.’ s paper, preferential sampling may be seen as a marked spatial point process model, in particular a marked log‐Gaussian Cox process. These models can be fitted in a computationally efficient way using integrated nested Laplace approximation (INLA) and associated software Rue, Martino, and Chopin (2009) in a fast computational way.

In this study, we thoroughly explain the methodology for performing preferential sampling models using the approach proposed by Rue et al. (2010) within the context of SDMs, with the final aim to provide guidance on the appropriate use and interpretation of the fitted models. A practical application assessing the spatial distribution of blue and red shrimp (*Aristeus antennatus, Risso 1816*) from fishing data in the Gulf of Alicante (Spain) is provided as a tutorial, while simulated data are used to demonstrate performance issues of standard methods which do not account for preferential sampling.

## 2 MATERIAL AND METHODS

Preferentially collected data consist of two pieces of information: (a) sampling locations, and (b) measured abundance (or occurrence) of target species in these locations, where the intensity of sampling locations is positively or negatively correlated with abundance, that is, (a) and (b) are not independent. Predicting the distribution of target species using this type of data implies that the sampling distribution is not uniformly random as it tends to have more observations where abundance is higher, and thus, basic statistical model assumptions are violated.

In order to overcome this problem, Diggle et al. (2010) proposed to interpret the preferential sampling process as a *marked point processes model*. This approach uses the information on sampling locations and models them along with the measured values of the variable of interest in a joint model. In particular, sampling locations are interpreted as a *spatial point pattern*, accounting for a higher point intensity where measured values are higher. The measurements taken in each of the points (the *mark* in point process terminology) are modeled along with and assumed to be potentially dependent on the point‐pattern intensity in a joint model. This approach accounts for preferential sampling while still making predictions for the variable of interest in space. In particular, in the fishery example discussed here, fishing locations are interpreted as a point pattern, while the species catch at each location is interpreted as a mark.

### 2.1 Statistical model

Formally, a spatial point pattern consists of the spatial locations of events or objects in a defined study region Illian, Penttinen, Stoyan, and Stoyan (2008). Examples include locations of species in a particular area, or parasites in a microbiology culture. Spatial point processes are mathematical models (random variables) used to describe and analyze these spatial patterns. A simple theoretical model for a spatial point pattern is the Poisson process, usually described in terms of its intensity function *Λ*_*x*_. This intensity function represents the distribution of locations (“points”) in space. In a Poisson process, the number of points follows a Poisson distribution and the locations of these points independent of any of the other points. The homogeneous Poisson process represents complete spatial randomness and serves as a reference or null model in many applications.

The intensity of a point process, that is, the number of points per unit area, may either be constant over space, resulting in a homogeneous or stationary pattern, or vary in space with a spatial trend, resulting in a non‐homogeneous pattern. However, the assumption of stationarity is generally unrealistic in most SDM applications as the intensity function varies with the environment, making non‐homogeneous Poisson processes potentially a better choice to describe species distribution based on a trend function that may depend on covariates. Nonetheless, in applications covariates may not explain the entire spatial structure in a spatial pattern. In contrast, the class of Cox processes provides the flexibility to model aggregated point patterns relative to observed and unobserved abiotic and biotic mechanisms. Here, spatial structures in an observed point pattern may reflect dependence on known and measured covariates, as well as on unknown or unmeasurable covariates or biotic mechanisms that cannot be readily represented by a covariate, such as dispersal limitation. Indeed, the spatial structure of both abiotic and biotic variables can impact on ecological processes and consequently be reflected in the species distribution.

Log‐Gaussian Cox processes (LGCPs) are a specific class of Cox process models in which the logarithm of the intensity surface is a Gaussian random field. Given the random field, More formally:

(1) $$\text{log}\left(\Lambda_{s}\right)=V_{s},$$

where *V*_*s*_ is a Gaussian random field. Given the random field, the observed locations ***s*** = (*s*_1_, …, *s*_*n*_) are independent and form a Poisson process.

In the case of preferentially sampled species, the observed abundance ***Y*** = (*y*_1_, …, *y*_*n*_) is also linked to the intensity of the underlying spatial field. In practice, only very few samples might be available in some areas if it is assumed that abundance is particularly low in these areas. The LGCP model fitted to the sampling locations reflects areas with low species abundance that have resulted in areas with fewer sampling locations. To incorporate such information in the SDM abundance model we apply joint modeling techniques, which allow fitting shared model components in models with two or more linear predictors. Here, we consider two dependent predictors with two responses, that is, the observed species abundances (the marks) and the intensity of the point process reflecting sampling intensity through space.

This results in a preferential sampling model that consists of two levels, where information is shared between the two levels, the mark model and the pattern model. In particular, the mark ***Y*** is assumed to follow an exponential family distribution such as a Gaussian, lognormal or gamma distribution for continuous variables or a Poisson distribution for count data. In all these cases, the mean *μ*_*s*_ is related to the spatial term through an appropriate link function *η*:

(2) $$\eta \left(\mu_{s}\right)=\beta_{0}^{\prime }+\beta_{n}^{\prime }X_{n}+W_{s},$$

where $\beta_{0}^{\prime }$ is the intercept of the model, the coefficients $\beta_{n}^{\prime }$ quantify the effect of some covariates *X*_*n*_ on the response, and *W*_*s*_ is the spatial effect of the model, that is, the Gaussian random field. The covariates in the additive predictor are environmental features linked to habitat preferences of a species.

In the second part of the model, an LGCP model with intensity function *Λ*_*s*_ reflecting the sampling locations:

(3) $$\Lambda_{s}=\text{exp}\left\{\beta_{0}+\beta_{n}X_{n}+\alpha W_{s}\right\},$$

where *β*_0_ is the intercept of the LGCP, the coefficients *β*_*n*_ quantify the effect of some covariates *X*_*n*_ on the intensity function, and *W*_*s*_ is the spatial term shared with the LGCP but scaled by α to allow for the differences in scale between the mark values and the LGCP intensities.

Bayesian inference turns out to be a good option to fit spatial hierarchical models because it allows both the observed data and model parameters to be random variables ([Banerjee, Carlin, & Gelfand, 2004), resulting in a more realistic and accurate estimation of uncertainty. An important issue of the Bayesian approach is that prior distributions must be assigned to the parameters (in our case, *β*_0_ and $\beta_{0}^{\prime }$) and hyperparameters (in our case, those of the spatial effect *W*) involved in the models. Nevertheless, as usual in this kind of models, the resulting posterior distributions are not analytically known and so numerical approaches are needed.

### 2.2 Fitting models with INLA

Model‐fitting methods based on Markov chain Monte Carlo (MCMC) can be very time‐consuming for spatial models, in particular LGCPs. Nevertheless, LGCPs are a special case of the more general class of latent Gaussian models, which can be described as a subclass of structured additive regression (STAR) models, (Fahrmeir & Tutz, 2001). In these models the mean of the response variable is linked to a structured predictor, which can be expressed in terms of linear and non‐linear effects of covariates. In a Bayesian framework, by assigning Gaussian priors to all random terms in the predictor, we obtain a latent Gaussian model. As a result, we can directly compute LGCP models using Integrated nested Laplace approximation (INLA). INLA provides a fast, yet accurate approach to fitting latent Gaussian models and makes the inclusion of covariates and marked point processes mathematically tractable with computationally efficient inference (Illian et al., 2013; Simpson, Illian, Lindgren, Sørbye, & Rue, 2016).

### 2.3 Preferential and non‐preferential models

As mentioned above, the resulting preferential model can be expressed as a two‐part model as follows. Assuming that the observed locations ***s*** = (*s*_1_, …, *s*_*n*_) come from a Poisson process with intensity $\Lambda_{s}=\text{exp}\left\{\beta_{0}+\alpha W_{s}\right\}$, we have to assign a distribution for the abundance. Based on the fact that the abundance is usually a positive outcome, we have considered a gamma distribution, although clearly other options could be possible (exponential, lognormal, or Poisson for counts). This yields:

(4) $$\begin{array}{cc} Y_{s} & ∼\text{Ga}\;(\mu_{s},\rho )\\ \text{log}\;(\mu_{s}) & =\beta_{0}^{\prime }+W_{s}\\ W & ∼N\;(0,Q\;(\kappa ,\tau ))\\ \left(2\log\kappa ,\log\tau \right) & ∼\text{MN}\;(\mu_{w},\rho_{w}) \end{array}$$

where the Gaussian random field *W*_*s*_ links the LGCP and the abundance process scaled by α in the Poisson process predictor to allow for differences in scale. The matrix Q(κ,τ) is estimated implicitly through an approximation of the Gaussian random field through the stochastic partial differential equation (SPDE) approach as in Lindgren, Rue, and Lindström (2011) and Simpson et al. (2016).

It is worth noting that not taking account of preferential sampling leads to biased results. This can be easily seen by comparing the preferential sampling approach with the following simpler model:

(5) $$\begin{array}{cc} Y_{s} & ∼\text{Ga}(\mu_{s},\rho )\\ \text{log}(\mu_{s}) & =\beta_{0}^{\prime \prime }+Z_{s}\\ Z & ∼N(0,Q(\kappa ,\tau ))\\ \left(2\log\kappa ,\log\tau \right) & ∼\text{MN}(\mu_{z},\rho_{z}) \end{array}$$

Note that the model in Equation (5) assumes that for the random fields we have Z≠W, whereas the preferential sampling model assumes a single, shared random field for both the point pattern and the mark. For both models, prior distributions have to be chosen for to all the parameters and hyperparameters. We have assigned vague priors, that is, used the default in R‐INLA due to a general lack of prior information. Another approach that could be used here is penalized complexity priors (hereafter pc.priors) as described in Fuglstad, Simpson, Lindgren, and Rue (2017) and readily available in R‐INLA.

As mentioned above, both models in (4) and (5) may include covariates, the significance of which may be tested through model selection procedures. There is very little literature available on model selection for point process models; however, criteria like the deviance information criterion (DIC) (Spiegelhalter, Best, Carlinm, & Van Der Linde, 2002) are sometimes used.

### 2.4 Simulated example

**Figure 1:**
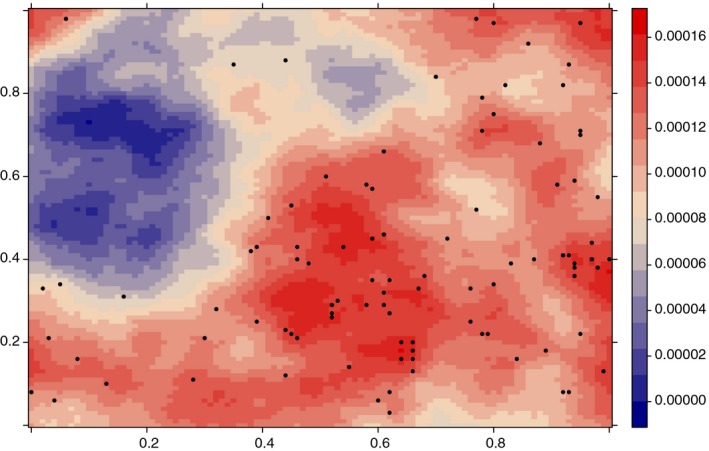
Representation of one of the one hundred Gaussian field simulated and the respective preferentially sampling locations generated

In order to illustrate the effectiveness of the preferential sampling method and to emphasize the misleading results we would obtain if we do not take it into consideration, we initially consider a simulation study (Figure 1). One hundred realizations of a Gaussian spatial random field with Matérn covariance function were generated over a 100‐by‐100 grid using the *RandomFields* package (Schlather, Malinowski, Menck, Oesting, & Strokorb, 2015).

For each of the 100 simulated Gaussian spatial random fields that represent the distribution of a species in the study area, two sets of 100 samples were reproduced, one distributed preferentially and one distributed randomly. In particular, preferentially locations were selected using relative probabilities proportional to the intensity function *Λ*_*s*_. Abundance estimates were extracted at the selected locations, as a simulation of the observations for this experiment.

Both preferential and non‐preferential models were fitted to each of the sample sets and compared in their performance using three different criteria: the deviance information criterion (DIC) (Spiegelhalter et al., 2002), the log‐conditional predictive ordinates (LCPO) (Roos & Held, 2011), and the predictive mean absolute error (MAE) (Willmott & Matsuura, 2005). Specifically, the DIC measures the compromise between the fit and the parsimony of the model, the LCPO is a “leaveoneout” cross‐validation index to assess the predictive power of the model, and the MAE indicates the prediction error. Lower values of DIC, LCPO and MAE suggest better model performance. Finally, a sensitivity analysis with different pc.priors was carried out to assess their influence on the final inference of the range and variance of a simulated Gaussian spatial random field.

### 2.5 Spatial distribution of blue and red shrimp in the Western Mediterranean Sea

In this section, we illustrate how preferential sampling can be accounted for in the concrete data example from the context of fishery data. Fishery‐dependent data derived from opportunistic sampling on boats from the commercial fleet present a standard example of preferential sampling, since clearly fishers preferentially fish in areas where they expect to find large amounts of their target species.

**Figure 2:**
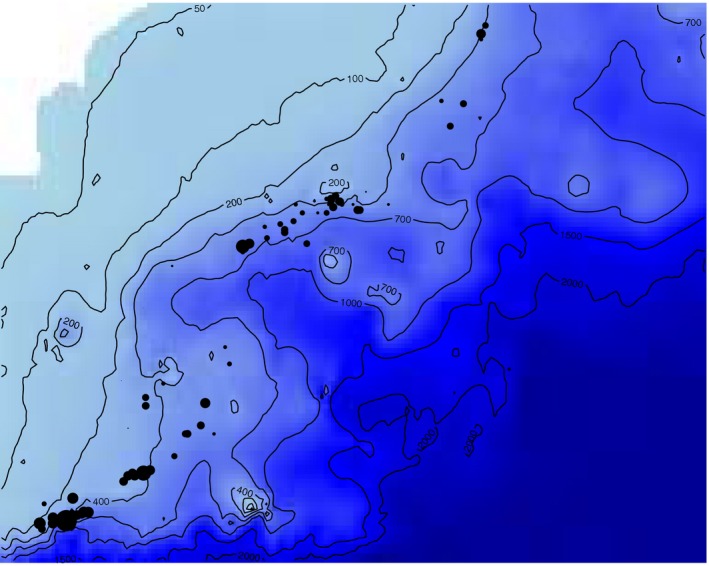
Study area and sampling locations (hauls) of blue and red shrimp (*Aristeus antennatus*). The size of the dots represents the amount caught in each of the locations

We consider data on blue and red shrimp (*Aristeus antennatus, Risso 1816*) (Carbonell et al., 2017; Deval & Kapiris, 2016; Lleonart, 2005), one of the most economically important deep‐sea trawl fishery in the Western Mediterranean Sea. The data were collected by observers onboard a number of fishing boats in the Gulf of Alicante (Spain) from 2009 to 2012. The dataset includes 77 hauls from nine different trawling vessels (Figure 2) and was provided by the *Instituto Español de Oceanografía* (IEO, Spanish Oceanographic Institute).

As mentioned, the fitted effects for the abundance of blue and red shrimp (*Y*) are corrected by jointly fitting a model for the abundances and the sampling (fishing) locations, reflecting fishers’ (potentially) imprecise knowledge of the distribution of blue and red shrimp. We also assume that the observed locations ***s*** = (*s*_1_, …, *s*_*n*_) come from a Poisson process with intensity $\Lambda_{s}=\text{exp}\left\{\beta_{0}+\alpha_{d}f\left(d\right)+\alpha_{w}W_{s}\right\}$, where *f*(*d*) represents the not necessarily linear relationship with one covariate, in this case bathymetry. The reason underneath this selection was that exploratory analysis revealed non‐linear relationships between depth and blue and red shrimp abundance. The remaining second part of the model, that is, the one explaining the abundance, also contains the relationship with the bathymetry:

(6) $$\begin{array}{cc} Y_{s} & ∼\text{Ga}(\mu_{s},\rho )\\ \text{log}(\mu_{s}) & =\beta_{0}^{\prime }+f\left(d\right)+W_{s}\\ W & ∼N(0,Q(\kappa ,\tau ))\\ \left(2\log\kappa ,\log\tau \right) & ∼\text{MN}(\mu_{w},\rho_{w})\\ \Delta d_{j}=d_{j}-d_{j+1} & ∼N(0,\rho_{d}),\;j=1,\ldots ,m\\ \rho_{d} & ∼\text{LogGamma}(4,0.0001) \end{array}$$

where *s* indexes the location of each haul and *j* indexes different depths (*d*_*j*_, representing the different values of bathymetry observed in the study area from *d*_1_ = 90 m tp *d*_*m *= 40_ = 920 m).

We use a Bayesian smoothing spline Fahrmeir and Lang (2001) to model non‐linear effects of depth, using a second‐order random walk (RW2) latent model.

As no prior information on the parameters of the model was available, we used a vague zero‐mean Gaussian prior distribution with a variance of 100 for the fixed effects. Regarding the spatial effect, priors on *μ*_*κ*_ and *μ*_*τ*_ were selected so that the median prior range of this component was half of the study area and its prior median standard deviation was 1. It was only in the case of the bathymetry that a visual pre‐selection of priors was made, to avoid overfitting, by changing the prior of the precision parameter while the models were scaled to have a generalized variance equal to 1 (Sørbye & Rue, 2014). In any case, all resulting posterior distributions concentrated well within the support of the priors selected.

Note that each predictor has its own intercept ($\beta_{0},\beta_{0}^{\prime }$) but bathymetric *f*(*d*) and spatial effects *W*_*s*_ are shared in both predictors. Also, both the bathymetric and the spatial effects are scaled by *α*_*d*_ and *α*_*w*_, respectively, to allow for the differences in scale between blue and red shrimp abundances and the LGCP intensities.

It is also worth noting that *ρ* is a parameter of the entire model that reflects the global variability of the response variable. As already mentioned before, in a preferential sampling situation, observations are more frequent where abundance values are likely to be higher and this fact could affect the inference about the ρ parameter. In line with this, a possible extension to our global modeling approach could be a model that could take into account this type of variability by linking ρ to the abundance measurements.

Model comparison was performed using the DIC and LCPO criteria. Finally, in order to test the prediction performance of the final preferential and non‐preferential models, we calculated the Pearson correlation (*r*) index between the predicted abundance estimates and an external database of observed abundance values in the same time period (2009–2012). This independent dataset includes fishery‐independent data collected during the MEDITS (EU‐funded MEDIterranean Trawl Survey). The MEDITS is carried out from spring to early summer (April to June) every year in the area, and it uses a random sampling (Pennino et al., 2016).

## 3 RESULTS

### 3.1 Simulated example

Results obtained in this simulation study show that not taking into consideration preferential sampling leads to misleading results, specially at low‐abundance areas.

**Figure 3:**
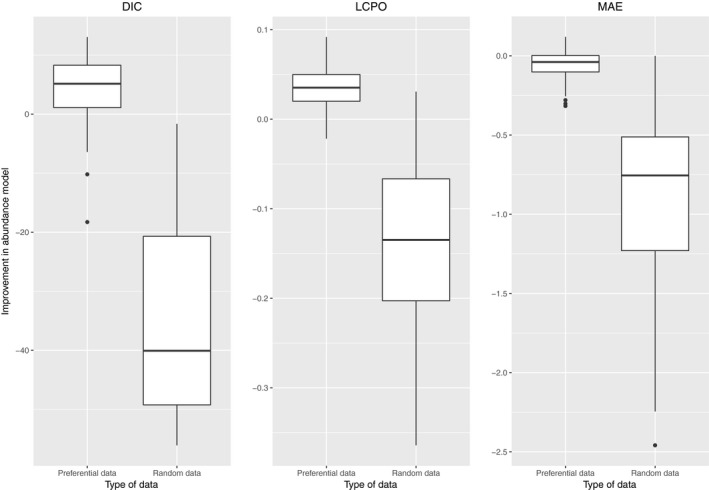
Improvement of the preferential model against a conventional model in model fit scores (DIC, LCPO, and MAE). Comparison is based on 100 preferentially and randomly sampled datasets. Note that positive values represent an improvement on model fit and vice versa

Figure 3 shows the difference in DIC, LCPO, and MAE scores between preferential and non‐preferential models for both samples, the ones distributed preferentially and the one distributed randomly. In particular, more than 75% of the DIC, LCPO, and MAE values obtained in the 100 simulated Gaussian fields using preferential models were lower than the ones obtained with non‐preferential models.

**Figure 4:**
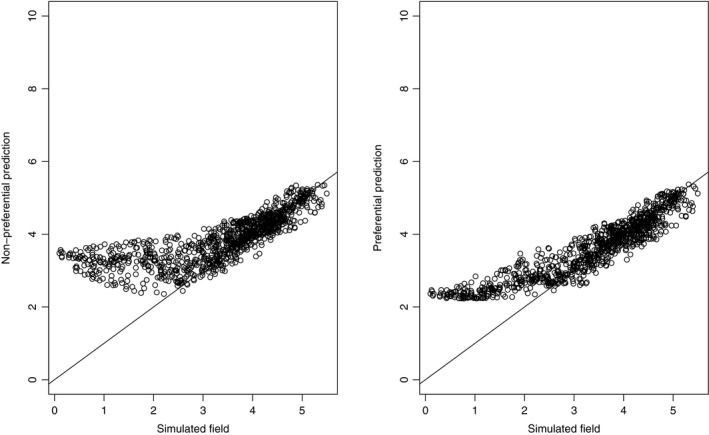
Simulated abundance against predicted abundance in the non‐preferential model (left) and in the model with the preferential correction (right) for one of the one hundred simulations performed. The non‐preferential model predicts worse than the preferential model at low‐abundance areas

In addition, as it can be appreciated in the example shown in Figure 4, which presents the results of one of the one hundred simulations for explanatory purposes, even if none of the models was able to make optimal predictions at low abundance locations, the non‐preferential model performed significantly worse.

**Figure 5:**
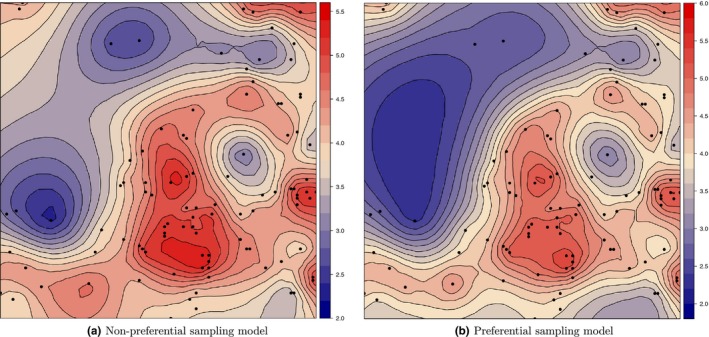
Posterior predictive mean maps of one of the one hundred simulated abundance processes without (left) and with (right) the preferential sampling correction

Similarly, Figure 5, which shows the posterior predictive mean of one of the simulated abundance processes without and with the preferential sampling correction ((a) and (b), respectively)), illustrates that although both models have similar predictive spatial patterns, the preferentially corrected model predicts better at moderate‐to‐low abundance areas.

**Figure 6:**
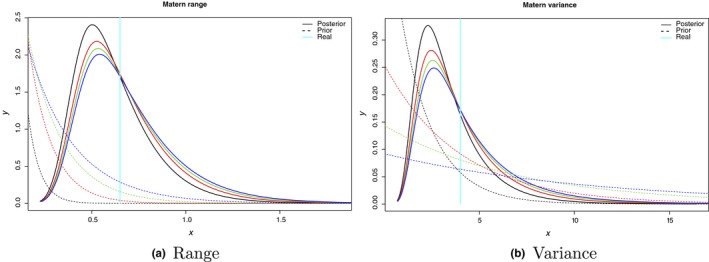
Sensitivity analysis of the pc.prior distributions for the range and variance of a simulated spatial field. Dashed lines represent prior distributions, solid lines posterior distributions and vertical lines the real values of each of the hyperparameters of the spatial field: range in the left panel and variance in the right panel. Range priors were set so that the probability of having a range smaller than 10%, 20%, 30%, 40% and 50% of the maximum distance of the study area was 0.25. Similarly, priors over the variance of the spatial field were set so that the probability of having a variance higher than 2, 3, 4, 5 and 6 was 0.1

Finally, Figure 6 shows how all different priors of the hyperparameters of the spatial field end up fitting posterior distributions that are all concentrated within the real values. Results clearly indicate that slight changes in the priors are not an issue when dealing with the preferential sampling models.

### 3.2 Distribution of blue and red shrimp in the Western Mediterranean Sea

All possible models derived from (6) were run. Among them, the most relevant results are presented in Table 1. While analyzing the data, we observed that both the bathymetric and the spatial terms of the LGCP accounted for approximately the same information. As a consequence, full models did not converge in the point‐pattern process, which restricted the model comparison in Table 1 to correcting only one of the effects, either the bathymetric or the spatial effect.

**Table 1:** Model comparison for the abundance of the blue and red shrimp (*Aristeus antennatus*) based on DIC, LCPO and computational times

	Model	DIC	LCPO	Times (s)
1	Intc + Depth	+19	+0.05	3
2	Intc + Spatial	+9	−	24
3	Intc + Depth + Spatial	+11	+0.01	57
4	Intc + **Depth**	+52	+0.24	21
5	Intc + **Spatial**	−	−	171
6	Intc + **Spatial** + Depth	+5	+0.02	212
7	Intc + **Depth** + Spatial	+10	+0.01	2,275
8	Intc + **Depth** + **Spatial**	+3	+0.03	3,470

Notes

DIC and LCPO scores are presented as deviations from the best model. Intc: Intercept; **Bold** terms: shared components.

The best model (based on the DIC and LCPO) was the preferential one with an shared spatial effect (i.e., Model 5 in Table 1). The second most relevant model in term is DIC, and LCPO was the preferential one that included, in addition to the spatial effect, a shared bathymetric effect (i.e., Model 8 in Table 1).

**Figure 7:**
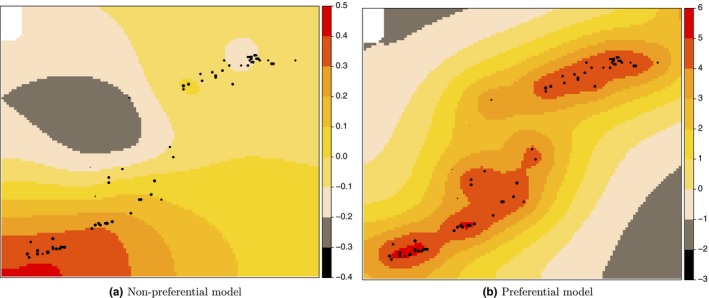
Maps of the mean of the posterior distribution of the spatial effect in the model without (left) and with (right) preferential sampling. Black dots represent sampling locations

**Figure 8:**
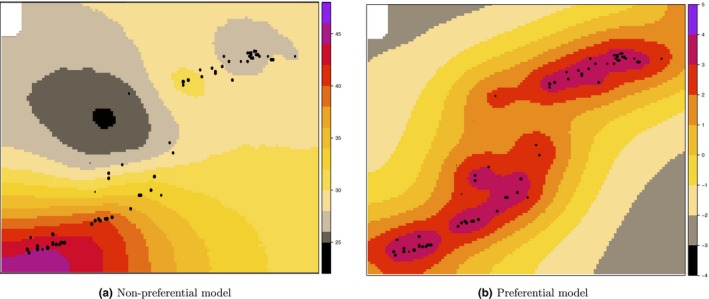
Posterior predictive mean maps of the blue and red shrimp (*Aristeus antennatus*) species, without and with the preferential sampling correction. Black dots represent sampling locations

Figure 7 shows the mean of the posterior distribution of the spatial effect in the model without and with preferential sampling, while Figure 8 illustrates the posterior predictive mean of the blue and red shrimp distribution without and with the preferential correction. Both figures show a similar pattern. Indeed, it is clear that the spatial outputs obtained with the preferential model better absorb the variability of the species habitat providing a more natural pattern of the blue and red shrimp distribution.

Finally, for model validation, the final preferential model obtained a reasonably high value for Pearson's *r* (*r* = 0.47) in the cross‐validation with the MEDITS dataset with respect to the non‐preferential one (*r* = 0.22). It is worth mentioning that MEDITSs are performed only in spring/summer and few samplings are carried out in the study area. These findings further highlight how the correction of the preferential model is important to reflect the real distribution of a species.

## 4 DISCUSSION

In this paper, we presented a modeling approach that could be very useful for modeling the distribution of species using opportunistic data and acquiring in‐depth knowledge that could be essential for the correct management of natural resources. Spatial ecology has a direct applied relevance to natural resource management, but it also has a broad ecological significance. Although it may be complicated to define the boundaries of species habitats combined with an efficient management that recognizes the importance of such areas, this represents the first step toward facilitating an effective spatial management. However, as shown by our results, using a non‐accurate approach could culminate in misidentification of a species habitat and uncertain predictions and so in inappropriate management measures which can sometimes be irreversible.

The results of the practical application on blue and red shrimp, included here as a real world scenario, show that predictive maps significantly improve the prediction of the target species when the model accounts for preferential sampling. Indeed, it is known that the suitable bathymetric range of the blue and red shrimp in the Mediterranean sea is between 200 and 200 meters (Guijarro, Massutí, Moranta, & Díaz, 2008) that is reflected by the preferential model prediction map. In contrast the non‐preferential model prediction was not able to capture the real distribution of the species. In addition, if a management measure, such as creation of a marine conservation area, should be applied on the basis of the non‐preferential model, it is clear that it would not be appropriate. This could result in extremely large area being recommended for protection, which is usually difficult to implement in most contexts, especially given the social and economic relevance of fishing (Reid, Almeida, & Zetlin, 1999). Moreover, the results of the cross‐validation with an external independent dataset further highlighted how the correction of the preferential model is important to reflect the real distribution of a species.

Nevertheless, even if the preferential model improves the estimation of the bathymetric effect, new observations at deeper waters could further improve this relationship and better understand the blue and red shrimp distribution in this area (Gorelli, Sardà, & Company, 2016).

Similarly, the simulated example showed that not taking into account the preferential sampling model could lead to misleading results.

Consequently, we conclude that this approach could suppose a major step forward in the understanding of target fished species mesoscale ecology given that most of the available data today are fishery‐dependent data. In addition, using a non‐preferential model with opportunistic data in a SDM context is not correct as spatial SDMs assume that the selection of the sampling locations does not depend on the values of the observed species.

Another advantage is undoubtedly the use of INLA in this context, which might be a key geostatistical tool due to its notable flexibility in fitting complex models and its computational efficiency (Paradinas et al., 2015).

This modeling could be expanded to the spatiotemporal domain by incorporating an extra term for the temporal effect, using parametric or semiparametric constructions to reflect linear, non‐linear, autoregressive or more complex behaviors that could be very important to describe the distribution of a particular species. Moreover, although we presented a case study related to abundance data, this approach could be also extended for species presence–absence data that are more common when SDMs are performed. In particular, using occurrences the modeling framework will be the same as the one described in our study, but the Gaussian field will be an approximation of the probability of the species presence.

Finally, it is worth to be noting that, as shown by Howard, Stephens, Pearce‐Higgins, Gregory, and Willis (2014), even using coarse‐scale abundance data, large improvements in the ability to predict species distributions can be achieved over their presence–absence model counterparts. Consequently, where available, it will be better to use abundance data rather than presence–absence data in order to more accurately predict the ecological consequences of environmental changes.

## CONFLICT OF INTEREST

None Declared.

## AUTHOR CONTRIBUTIONS

All authors conceived and designed the work, drafted the work, and gave final approval of the version to be published. M.G.P., I.P., F.M., and J.I. analyzed the data.

## DATA ACCESSIBILITY

Data underlying this article are available on request: please contact graziapennino@yahoo.it.
